# The Potential Impact of Zinc Supplementation on COVID-19 Pathogenesis

**DOI:** 10.3389/fimmu.2020.01712

**Published:** 2020-07-10

**Authors:** Inga Wessels, Benjamin Rolles, Lothar Rink

**Affiliations:** ^1^Institute of Immunology, Faculty of Medicine, RWTH Aachen University, Aachen, Germany; ^2^Department of Hematology, Oncology, Hemostaseology and Stem Cell Transplantation, Faculty of Medicine, RWTH Aachen University, Aachen, Germany

**Keywords:** zinc, COVID-19, SARS-CoV2, 2019-nCoV, coronaviridae, zinc deficiency, impaired immune system

## Abstract

During the current corona pandemic, new therapeutic options against this viral disease are urgently desired. Due to the rapid spread and immense number of affected individuals worldwide, cost-effective, globally available, and safe options with minimal side effects and simple application are extremely warranted. This review will therefore discuss the potential of zinc as preventive and therapeutic agent alone or in combination with other strategies, as zinc meets all the above described criteria. While a variety of data on the association of the individual zinc status with viral and respiratory tract infections are available, study evidence regarding COVID-19 is so far missing but can be assumed as was indicated by others and is detailed in this perspective, focusing on re-balancing of the immune response by zinc supplementation. Especially, the role of zinc in viral-induced vascular complications has barely been discussed, so far. Interestingly, most of the risk groups described for COVID-19 are at the same time groups that were associated with zinc deficiency. As zinc is essential to preserve natural tissue barriers such as the respiratory epithelium, preventing pathogen entry, for a balanced function of the immune system and the redox system, zinc deficiency can probably be added to the factors predisposing individuals to infection and detrimental progression of COVID-19. Finally, due to its direct antiviral properties, it can be assumed that zinc administration is beneficial for most of the population, especially those with suboptimal zinc status.

## Introduction

The importance of the trace element zinc for the development and function of the immune system across all kinds of species has been proven in numerous studies (1–3). As zinc deficiency results in altered numbers and dysfunction of all immune cells, subjects with suboptimal zinc state have an increased risk for infectious diseases, autoimmune disorders, and cancer (3–6). In addition to malnutrition, risk groups for zinc deficiency include the elderly and patients with various inflammatory and autoimmune diseases, which will be discussed in detail later in the article (7, 8). As mild zinc deficiency is largely sub-clinical, it is unnoticed in most people. However, the World Health Organization (WHO) assumes that at least one third of the world population is affected by zinc deficiency (9). The fact that zinc deficiency is responsible for 16% of all deep respiratory infections world-wide (9) provides a first strong hint on a link of zinc deficiency with the risk of infection and severe progression of COVID-19 and suggests potential benefits of zinc supplementation.

**Figure 1:**
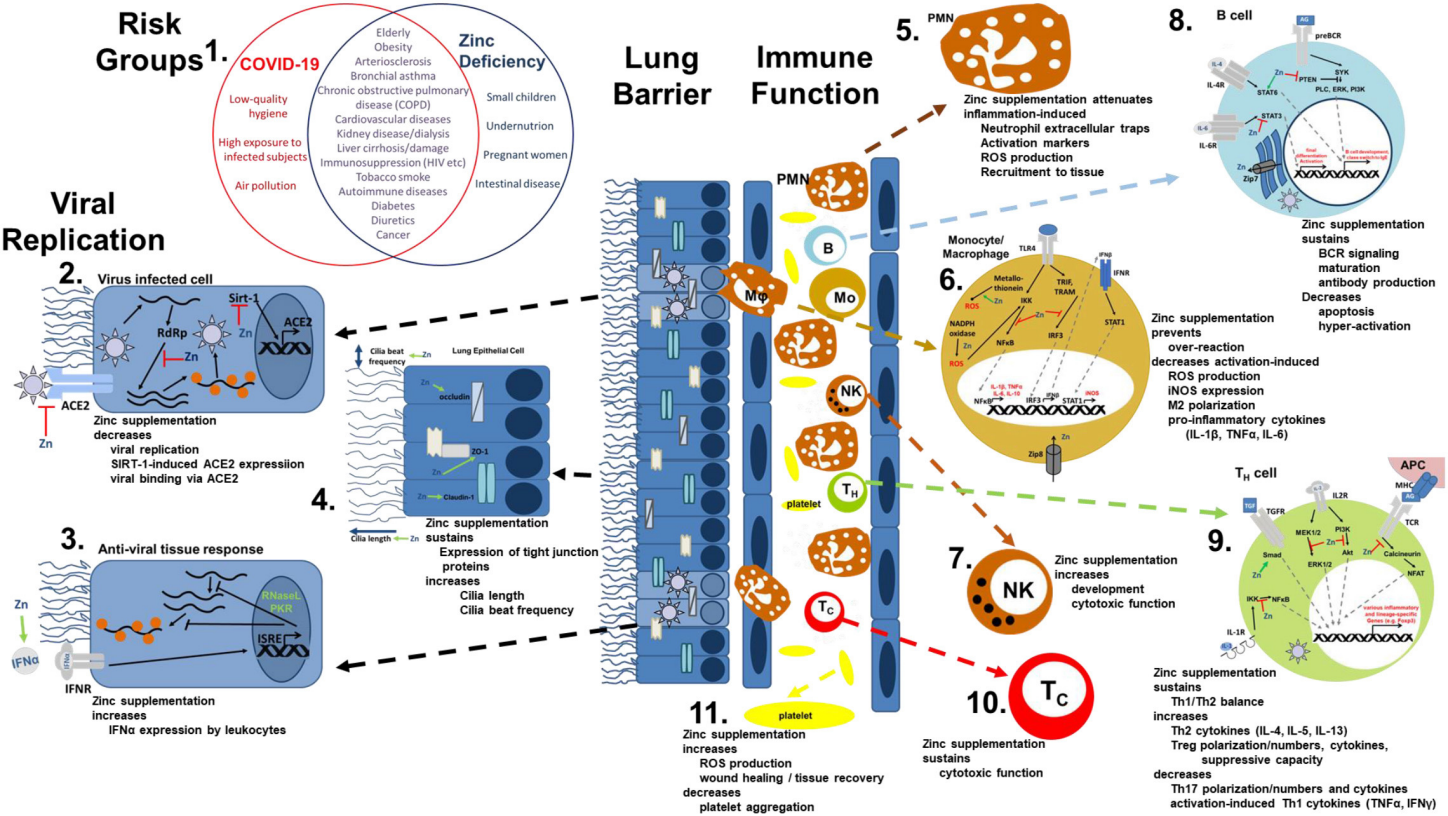
Viral mechanism of COVID-19 and how they might be opposed by zinc data. **(1)** There is an impressive intersection of known risk factors for zinc deficiency (blue circle) and the predisposition for a severe COVID-19 infection (red circle). **(2,3)** Zinc (Zn) supplementation might already prevent the viral entry and also suppresses its replication, while it supports the anti-viral response of the host cells. **(4)** As zinc is known to increase ciliary length and movements and also sustains tissue integrity, entrance of the virus is impeded. **(5−10)** The importance of zinc on the development and function of the immune cells is manifold. It should be underlined, that zinc's effects should not generally be described as activating or inhibiting, as zinc in various cases normalizes overshooting immune reactions and balances the ratios of the various immune cell types. Zinc thus prevents for example that high levels of inflammatory mediators including reactive oxygen and nitrogen species destroy the host tissue. **(11)** On first view it appears contradicting, that zinc increases activation induced production of reactive oxygen species in platelets, while it is generally considered as anti-oxidative. However, in case of platelets, up to a certain threshold, ROS production is essential, as it can prevent the formation of platelet aggregates. In summary, zinc therefore might be able to prevent vascular complications observed in COVID-19 patients. Details for each point can be found in the text. ACE2, angiotensin converting enzyme 2; AG, antigen; IFN, interferon; IFNR, interferon receptor; ISRE, interferon-sensitive response element; APC, antigen presenting cell; IKK, IκB kinase; IL, interleukin; iNOS, inducible nitric oxide synthase; IRF3, IFN regulatory factor 3; MHC, major histocompatibility complex; MEK1/2, mitogen-activated protein kinase kinase 1/2; NADPH oxidase, nicotinamide adenine dinucleotide phosphate oxidase; NFAT, nuclear factor of activated T-cells; NF-κB, nuclear factor kappa B; PKR, protein kinase R; Akt, protein kinase B; PI3K, phosphatidylinositol-3 kinases; ROS, reactive oxygen species; RdRP, RNA-dependent RNA polymerase; RNase L, ribonuclease L; Sirt-, Sirtuin 1; STAT, signal transducer and activators of transcription; TCR, T cell receptor; Tc, cytotoxic T cell; TH, helper T cell; TGF, transforming growth factor; TRAM, TRIF-related adaptor molecule; TRIF, TIR-domain-containing adapter-inducing interferon-β; TLR, toll-like receptor; TNF, tumor necrosis factor; Zip, Zrt- and Irt-like protein; ZO-1, zona occludens.

The most common symptoms of COVID-19 are impaired smell and taste, fever, cough, sore throat, general weakness, pain as aching limbs, runny nose, and in some cases diarrhea (10). In the subsequent chapters, we will associate most of those symptoms with altered zinc homeostasis and explain how zinc might prevent or attenuate those symptoms, as summarized in Figure 1, and thus should be regarded as promising cost-effective, globally available therapeutic approach for COVID-19 patients, for which minimal to no side effects are known.

## Zinc Protects the Human Body From Entering of the Virus

The entry of infectious agents into the human body is prevented by tissue barriers equipped with cilia and mucus, anti-microbial peptides like lysozymes and interferons. Regarding SARS-CoV2, the angiotensin-converting enzyme 2 (ACE2) and the cellular protease TMPRSS2 are the major mechanism for entering the cells (11).

- Mucociliar clearance of viruses is affected by zinc
  Infections with coronaviruses go along with damage of the ciliated epithelium and ciliary dyskinesia consecutively impairing the mucociliar clearance (12). It was shown that physiological concentrations of zinc increase ciliary beat frequency (13). Moreover, zinc supplementation in zinc deficient rats had a positive effect on the number and the length of bronchial cilia (14) (Figure 1.4). Improved ciliary clearance does not only improve the removal of virus particle, it also reduces the risk of secondary bacterial infections, as discussed later. Alterations of the extracellular matrix, as monitored by increased epidermal growth factor and proliferating cell nuclear antigen (PCNA) immunostaining of rat lungs during zinc deficiency have also been described (15).
- Zinc is essential for preserving tissue barriers
  Disturbances in the integrity of the respiratory epithelia facilitate the entry of the virus as well as co-infecting pathogens and can lead to pathogens entering the blood stream. An *ex-vivo* model of the chronic obstructive pulmonary disease (COPD) showed that decreasing zinc levels raised the leakage of the epithelium of the respiratory tract (16), while zinc supplementation improved lung integrity in a murine model of acute lung injury *in vivo* (17). Increased apoptosis and E-cadherin/beta-catenin proteolysis were amongst the underlying mechanisms (17–19). The expression of tight junction proteins like Claudin-1 and ZO-1 was found to be zinc-dependent, offering another explanation for zinc's positive effects on lung integrity (16). In addition, an inhibitory effect of zinc on LFA-1/ICAM-1 interaction weakened inflammation in the respiratory tract via reduction of leukocyte recruitment (20). Furthermore, high zinc levels improved the tolerance of the lung towards damage induced by mechanical ventilation (21) (Figure 1.4).
- Zinc-dependent alterations in gene expression by pneumocytes could affect viral entering
  ACE-2, mainly expressed on pneumocytes type 2, is a zinc-metalloenzyme. Zinc binds to its active center and is thus essential for its enzymatic activity. If zinc binding also affects the molecular structure of ACE-2 and thereby its binding affinity to the virus, remains to be tested (22, 23). However, this is likely as zinc is important for stabilizing protein structures and altering substrate affinity of various metalloproteins (24, 25). Finally, zinc homeostasis might affect ACE-2 expression, as zinc-dependent expression was reported for other zinc-metalloenzyme such as metallothionein and matrix metalloproteinases (26). This suggestion is strengthened by the finding that ACE-2 expression is regulated by Sirt-1 (27, 28). As zinc decreases Sirt-1 activity (27), it might decrease ACE-2 expression and thus viral entry into the cell (Figure 1.2).

A lack of adequate secretion of type I and type II interferons was reported for COVID-19 patients (29). For human interferon alpha (IFN-α) it was shown that zinc supplementation can reconstitute its expression by leukocytes and potentiates its anti-viral effect via JAK/STAT1 signaling as observed for rhinovirus-infected cells (30, 31). However, as it was suggested that SARS-CoV2 might take advantage of the interferon-dependent expression of ACE2, which was recently addressed by Ziegler et al. (32), the overall effects of zinc need to be carefully evaluated in future studies.

## Zinc Directly Inhibits Viral Replication

As a virus, SARS-CoV2 is highly dependent on the metabolism of the host cell. Direct antiviral effects of zinc have been demonstrated in various cases, which was reviewed in great detail (33–37). Examples include coronaviridae, picornavirus, papilloma virus, metapneumovirus, rhinovirus, herpes simplex virus, varicella-zoster virus, respiratory syncytial virus, human immunodeficiency virus (HIV), and the hepatitis C virus (34, 35, 37–39). It was suggested that zinc can prevent fusion with the host membrane, decreases the viral polymerase function, impairs protein translation and processing, blocks viral particle release, and destabilizes the viral envelope (35, 37, 40). Low-dose zinc supplementation together with small concentrations of the zinc ionophores pyrithione or hinokitol decreased RNA synthesis in influenza, poliovirus, picornavirus, the equine arteritis virus, and SARS-CoV by directly inhibiting the RNA-dependent RNA polymerase of the virus (34, 41). There is evidence that zinc can enhance the effect of chloroquine, another known zinc ionophore, while zinc ionophores like epigallocatechin-gallate or quercetin remain to be tested (42–45). There are close similarities of SARS-CoV2 and other coronaviridae like SARS-CoV and Middle East respiratory syndrome-related coronavirus (MERS-CoV) (46). Also, the alcohol-aversive drug disulfiram can bind the papain-like proteases of SARS-CoV and MERS-CoV resulting in release of cysteine-bound zinc that results in protein destabilization (47). Detailed studies on zinc's effect specifically on SARS-CoV2 are highly required (Figure 1.3).

## Zinc Balances the Immune Response During Infectious Diseases

One of the hallmarks of COVID-19 is an imbalanced immune response (48). Due to hyper-inflammation, immune products including pro-inflammatory cytokines like interleukin (IL)-6, C-reactive protein (CRP), tumor necrosis factor (TNF)α and IL-1β (summarized as cytokine storm or cytokine release syndrome), reactive oxygen, and nitrogen species in connection with the recruitment of high numbers of strongly activated immune cells to the lungs, the destruction of the tissue, permanent lung damage and death due to systemic inflammation, and organ failure are expected, while the anti-inflammatory response is insufficient (48–52). A high number of patients develop an acute respiratory distress syndrome (ARDS) accompanied by high alveolar leakage leading to alveolar and interstitial edema with severely limited oxygen exchange (53). Advanced SARS-CoV2 infections are characterized by a systemic involvement with organ complications and accompanying cytokine storm (52, 54).

There is no doubt on the anti-inflammatory and anti-oxidative properties of zinc and underlying mechanisms have been the focus of numerous studies (1–3, 6, 55–60). A detailed description of zinc metabolism in airway epithelium and during inflammation of the airways has been published by Zalewski et al. (61). On the other hand, zinc deficiency was associated with elevated levels of pro-inflammatory mediators, increased reactive oxygen species (ROS) levels and pre-disposing for severe progression of inflammatory diseases, especially those affecting the lung, often reversible by zinc supplementation (6, 17, 56, 62–66) (Figures 1.5,1.6). As one example, exposure to organic dust increased lung damage, inflammation and macrophage hyper-activation in animals with zinc deficiency, predisposing these animals to pulmonary fibrosis, while zinc supplementation 24 h before induction of acute lung injury significantly attenuated the inflammatory reaction and tissue damage (17, 67). Regarding systemic inflammatory diseases the number of studies showing benefits of especially preventive zinc supplementation is constantly increasing (17, 18, 58, 65, 68). Amongst the underlying mechanism, zinc's role as second messenger and importance in regulating intracellular signaling as detailed in Figure 1 were described as well as zinc's effects on the epigenome (56, 57, 69–74).

Furthermore, leukocytosis with neutrophilia and lymphopenia, especially affecting CD8^+^ T cells, were associated with poor prognosis of COVID-19 and the recovery of lymphocyte counts lead to clinical recovery (75, 76). Similar changes in lymphopoiesis and myelopoiesis have been described in zinc deficient rodents, which were normalized when zinc was supplemented (17, 19). Circulating and lung-resident T cells from COVID-19 patients showed increased expression of markers for T cell exhaustion like Tim-3 and PD-1 (77). The extent of these changes had an impact on the patient's prognosis (50). During the past decades, an immense literature was generated on the need of zinc for lymphocyte development and function and that zinc supplementation (6, 19, 63, 64, 78, 79) can reverse lymphopenia. Enumerating all findings and underlying mechanisms is beyond the scope of this article, and a lot of aspects have been discussed in related publications (36) but as one of the many key roles of zinc in the context of T cell function, zinc is indispensable in the signal cascade of the T cell receptor and IL-2 as a second messenger (78, 80) (Figure 1.9). The B cell compartment also strongly benefits from a balanced zinc homeostasis, as zinc is required for B cell maturation and function (72, 81) (Figure 1.8). Also important to mention, but neglected by previous related articles, is that there is evidence (82, 83) that SARS-CoV2 can directly infect T cells as well as B cells and impair their cell specific function. This could explain the impact of SARS-CoV2 infection on lymphoid tissues like the human spleen and lymph nodes (84). However, as data are limited to *in vitro* experiments, this needs to be verified *in vivo* as well as if zinc affects the virus-induced changes in T and B cells.

Additionally, granulocytes play a vital role during the inflammation-induced destruction of the lung (85). Recent evidence suggests that lipopolysaccharide-induced hyper-activation, recruitment and formation of neutrophil extracellular traps are attenuated by zinc supplementation *in vivo* and that cytokine expression, phagocytosis and burst, chemotaxis and degranulation, and intracellular signaling are zinc regulated (17, 86, 87) (Figure 1.5). Important defense mechanisms of the innate immunity include the toll like receptors. For instance, *in silico* data suggest that toll-like receptor (TLR)-4 can potentially recognize outer components of SARS-CoV2's like the viral spikes (88), while intracellular receptors including TLR3, TLR7/8, and TLR9 can recognize viral dsRNA, ssRNA, and unmethylated CpG DNA respectively (89–92). Intranasal pretreatment with a TLR3 agonist and, to a lesser extent, with TLR9, TLR7/8, or TLR4 agonists, provided a high level of protection against infections by SARS coronavirus and influence virus in mice, suggesting that TLR signaling can induce protective antiviral immunity (93). This might be a completely novel approach to consider regarding COVID-19 as well. Zinc is an essential regulator in TLR-4- and TLR-3-induced signaling in innate immune cells (94). Thus, zinc deficiency potentially disturbs the innate immune response toward SARS-CoV2, enabling the virus to easily spread throughout the host without an adequate immune response (Figure 1.6).

Clinical improvement of COVID-19 patients was correlated to an increase of CD14^+^ monocytes and NK cells in the recovery phase (48). For a physiological inflammatory response and phagocytic activity macrophages need sufficient intracellular zinc levels (1). In addition, for NK cells and cytotoxic T cells it was shown that zinc supplementation increased their cytotoxicity toward target cells (1, 2, 95) (Figures 1.7,1.10).

In summary zinc's (re-)balancing power regarding immune cell numbers and functions might be highly beneficial in regard to therapy of COVID-19.

## Zinc Supplementation in Respiratory Infections

Our suggested benefits of zinc supplementation to prevent and treat COVID-19 are supported by a row of successful supplementation studies focusing on respiratory tract infection, of which we listed some selected publications in Table 1. In most cases, prophylactic zinc supplementation was more effective than therapeutic proceedings (106–108, 111). Up to 30% of the everyday respiratory infections, briefly named “common cold,” are due to infections with coronaviruses (112). Studies showed reduced symptom severity, reduced frequency, and duration of the common cold after zinc administration (99, 100, 113, 114) depending on dosage, zinc compound and the start time after initial symptoms (115). Most importantly, zinc supplementation of children revealed significant benefits in various studies (96, 106) and reduced 15% pneumonia-specific morality and 19% of pneumonia morbidity in developing countries (116).

**Table 1:** Selected zinc supplementation studies in respiratory infections.

**Compound**	**Conc. [mg/d]**	**Duration**	**Disease**	**Effect**	**References**
**Treatment**					
Zinc bis-glycinate	30 (elemental)	Max 7 days/dis-charge from the hospital	Lower RTI (Children)	Reduction of days of ALRI and shorter hospital stay	(96)
Zinc acetate	20	5d	Lower RTI (children)	Increased recovery rates (boys)	(97)
Zinc gluconate	10	6 mo	Lower RTI (children)	Decreased episodes of infection, more infection free days	(98)
Zinc gluconate Zinc actetate Gluconate nasal gel SULFITE nasal spray	60–313 76.8–102.4 2.1 0.044	Until symptoms are gone	Common cold	Variable results but generally reduced duration if supplementation started within first 24 h	Meta-study of 16 studies (99)
Zinc acetate vs. zinc gluconate	80–92 192–207	Until symptoms are gone	Common cold	<75 mg/day: reduced duration; zinc acetate better than gluconate	Meta-study of 7 studies (100)
Zinc gluconate	30 (elemental)	12 mo	Cystic fibrosis (children)	Reduced duration of antibiotics	(101)
**Prophylactic**					
Zinc sulfate	20 (elemental)	2 wk/6 mo follow-up	Lower RTI (children)	Reduced morbidity	(102)
Zinc sulfate	20 to ZD children	14d, 6 mo follow-up	Upper and lower RTI (children)	Decreased incidence and duration of upper and lower RTI	(103)
Zinc oxide	5	12 mo	Upper RTI (children)	Decreased incidence	(104)
Zinc gluconate	10	6 mo	Lower RTI	Decreased incidence	(105)
Zinc acetate, gluconate, methionine, sulfate	Min 70 mg/wk	>3 mo	Lower RTI	Decreased incidence (depending on criteria)	Meta-study of 10 studies (106)
Zinc in mineral mix	6 (f)−7.5 (m)	12 mo	Naturally occurring pneumonia	Decreased incidence and duration, decreased duration of antimicrobial therapy	(107)
Zinc sulfate	60–90	12 mo	Ventilation associated pneumonia	Decreased incidence	(108)
Zinc gluconate	Up to 12x 23 mg/d	Until symptoms are gone	Common cold	Decreased clinical score	(109)
Zinc sulfate	15	7 mo	Common cold	Decreased incidence	(110)
**Murine models**					
Zinc-enriched rodent diet	ZD: 50 ppm–ZS: 100 ppm	18d ZD followed by 3d ZS	Sepsis-induced ALI	Decrease in inflammation, lung damage, and mortality (vs. ZD mice)	(68)
Zinc aspartate	30 μg/ mouse	24 h	Acute lung injury (LPS inhalation), mice	Decreased hyper-inflammation, tissue damage	(17)

*Conc, concentration; d, day; mo, months; ref, reference; RTI, respiratory tract infection; ZD, zinc deficiency; ZS, zinc supplementation; wk, week*.

*Single studies are not included in the meta-studies*.

## Risk Groups and Symptoms of COVID-19 and Zinc Deficiency Reveal a Large Overlap

As illustrated in Figure 1.1, the intersection between risk groups of COVID-19 and zinc deficiency is impressive. In patients with chronic obstructive pulmonary disease (COPD), bronchial asthma, cardiovascular diseases, autoimmune diseases, kidney diseases, dialysis, obesity, diabetes, cancer, atherosclerosis, liver cirrhosis, immunosuppression, and known liver damage low serum zinc levels are regularly observed (4, 117). At the same time, these groups are particular at risk for COVID-19 (10, 51, 118, 119). For example 57.5% elderly and nursing home residents in the U.S., for which high incidence of respiratory tract infections is described, showed significantly decreased zinc intake levels and are considered subjects with high risk regarding COVID-19 (120). Moreover, other studies showed that serum zinc levels were inversely correlated with pneumonia and cystic fibrosis (121, 122). On the other hand, zinc supplementation was able to reconstitute immune function in elderly and zinc deficient individuals (107, 123), which remains to be addressed for SARS-CoV2 infections (36). In this regard, the low response of older patients with low serum zinc to a 23-valent pneumococcal polysaccharide vaccination compared to those with higher zinc level (124), should be mentioned. However, zinc's role in the response to vaccination is generally discussed controversially and no data are available for vaccination against any corona virus.

Several studies indicate that there is an association between chemosensory dysfunctions and COVID-19 (125–133). Smell or taste is largely decreased, which might be a good disease biomarker (133). It was suggested that this might either be due to direct destruction of sensory cells by the virus, as ACE-2 is highly expressed by the oral mucosa, or by viral entry into the brain and neuronal pathologies as was described for other SARS-CoV (133, 134). Zinc deficiency was related to significantly reduced taste sensitivity and impaired saliva secretion in humans and animals, which might involve zinc's importance for the action of carbonic anhydrase (135–140). Results from supplementation studies largely describe improvements in chemosensory functions (140, 141), but some studies did not find any effects (142) or even more severe disturbances when very high zinc concentrations were used (143). This is possibly due to investigating olfactory diseases of various origins, the lack of controlled trials and inclusion of observable studies. Thus, the benefits of zinc supplementation alone or in combination with common medical strategies should be tested for taste and smell diseases according to the available guidelines (144).

About 50% of patients that died of COVID-19 had bacterial or fungal co-infections (145), underlining the importance of sustaining the immune function by a sufficient zinc supply (1, 2, 36). In animal experiments it was shown that zinc restriction made mice highly susceptible to bacterial infection with *streptococcus pneumoniae* (146). As mentioned earlier, marginal zinc deficiency affects one third of the worldwide population and most subjects with COVID-19 are at risk of zinc deficiency (Figure 1). During physiological inflammatory responses, zinc is additionally redistributed to the tissues, resulting in serum hypozincemia (1, 65). In combination with the pre-existing suboptimal zinc supply, this will decrease serum zinc levels to critically low values and thereby significantly increase the susceptibility for co-infections with detrimental progression. In critically ill patients persistent low serum zinc was associated with recurrent sepsis and serum zinc levels were inversely correlated with mortality from sepsis (62), underlining the potential benefits of monitoring the zinc status of the patients and implementing zinc supplementation into therapy of COVID-19.

Vascular complications resulting from atherosclerosis development, microangiopathic organ failure, and venous thromboembolism were found as a major cause of death in COVID-19 patients (147–149), suggesting an important role of disease-induced coagulopathy, which, however, needs further investigation. Zinc influences thrombocyte aggregation and coagulation (150). Recently, a functional association between zinc and ROS production in platelets was described, indicating that zinc could decrease thrombus formation in a clinical context (151). Complications of SARS-CoV2 infections also include tissue damage affecting the gastrointestinal system (152), the liver (153), heart (154), nervous system (155), kidneys (156), blood vessels (149), and the skin (157). In this regard it should be mentioned that a balanced zinc homeostasis is essential for wound healing and tissue recovery after mechanical and inflammation-mediated damage (158, 159), adding more potential benefits of zinc supplementation of COVID-19 patients (Figure 1.11).

## Conclusion

In this perspective, we reviewed the most important literature on the role of zinc homeostasis during viral infections, focusing on the potential benefits of zinc supplementation to prevent and treat SARS-CoV2 infections. Although data specifically on SARS-CoV2 are unfortunately still pending and randomized controlled studies have not been conducted, the enumerated evidence from the literature strongly suggests great benefits of zinc supplementation. Zinc supplementation improves the mucociliary clearance, strengthens the integrity of the epithelium, decreases viral replication, preserves antiviral immunity, attenuates the risk of hyper-inflammation, supports anti-oxidative effects and thus reduces lung damage and minimized secondary infections. Especially older subjects, patients with chronic diseases and most of the remaining COVID-19 risk groups would most likely benefit. Although studies are needed testing the effect of zinc as therapeutic option for established disease, preventive supplementation of subjects from risk groups should begin now, as zinc is a cost-efficient, globally available and simple to use option with little to no side effects. The first clinical trials on zinc supplementation alone and in combination with other drugs such as chloroquine have been registered (124, 160–162). Thus, first results and treatment regimens regarding zinc supplementation for COVID-19 risk groups and patients can be anticipated soon.

## Data Availability Statement

The original contributions presented in the study are included in the article/supplementary material, further inquiries can be directed to the corresponding author/s.

## Author's Note

LR is a member of ZINC Net.

## Author Contributions

IW, BR, and LR drafted and corrected the text, table, and figure. All authors contributed to the article and approved the submitted version.

## Conflict of Interest

The authors declare that the research was conducted in the absence of any commercial or financial relationships that could be construed as a potential conflict of interest.
